# ARF6 is a host factor for SARS-CoV-2 infection *in vitro*

**DOI:** 10.1099/jgv.0.001868

**Published:** 2023-06-21

**Authors:** C. Mirabelli, J. Bragazzi Cunha, J. W. Wotring, E. J. Sherman, J. El Saghir, J. Harder, M. Kretzler, J. Z. Sexton, B. T. Emmer, C. E. Wobus

**Affiliations:** ^1^​ Department of Microbiology and Immunology, University of Michigan Medical School, Ann Arbor, Michigan, USA; ^2^​ Department of Internal Medicine, Division of Hospital Medicine, University of Michigan, Ann Arbor, Michigan, USA; ^3^​ Department of Internal Medicine, Division of Gastroenterology and Hepatology, University of Michigan, Ann Arbor, Michigan, USA; ^4^​ Department of Internal Medicine, Division of Nephrology, University of Michigan, Ann Arbor, Michigan, USA; ^†^​ Present address: Institut für Virologie und Zellbiologie, University of Lübeck, Lübeck, Germany; ^‡^​ Present address: Territory Manager at Takara Bio, Inc., MI, MN, IN, KY, USA

**Keywords:** ARF6, endocytic pathway, entry, SARS-CoV-2, therapeutic targets

## Abstract

Severe acute respiratory syndrome coronavirus 2 (SARS-CoV-2) is a newly emerged beta-coronavirus that enter cells via two routes, direct fusion at the plasma membrane or endocytosis followed by fusion with the late endosome/lysosome. While the viral receptor, ACE2, multiple entry factors and the mechanism of fusion of the virus at the plasma membrane have been investigated extensively, viral entry via the endocytic pathway is less understood. By using a human hepatocarcinoma cell line, Huh-7, which is resistant to the antiviral action of the TMPRSS2 inhibitor camostat, we discovered that SARS-CoV-2 entry is not dependent on dynamin but on cholesterol. ADP-ribosylation factor 6 (ARF6) has been described as a host factor for SARS-CoV-2 replication and is involved in the entry and infection of several pathogenic viruses. Using CRISPR/Cas9 genetic deletion, a modest reduction in SARS-CoV-2 uptake and infection in Huh-7 was observed. In addition, pharmacological inhibition of ARF6 with the small molecule NAV-2729 showed a dose-dependent reduction of viral infection. Importantly, NAV-2729 also reduced SARS-CoV-2 viral loads in more physiological models of infection: Calu-3 cells and kidney organoids. This highlighted a role for ARF6 in multiple cell contexts. Together, these experiments point to ARF6 as a putative target to develop antiviral strategies against SARS-CoV-2.

## Introduction

Severe acute respiratory syndrome coronavirus 2 (SARS-CoV-2) is a newly emerged beta-coronavirus and causative agent of the coronavirus disease 2019 (COVID-19) pandemic. While several vaccines and antivirals have been approved to limit severe COVID-19, the spread of the virus is not yet under control. This highlights the importance of developing additional therapeutic strategies that can complement vaccine rollout, especially at the epidemic centres. Given the propensity of RNA viruses to develop drug resistance, there is an unmet need for additional antiviral compounds that can curb infection and transmission by targeting additional steps in the virus life cycle.

Viral entry is a step in the viral cycle that is promising for antiviral development. This important step of the viral life cycle was characterized very early after the isolation of SARS-CoV-2, leading to the discovery of the uncoating receptor, ACE2 [1], and the two entry pathways [2], plasma membrane fusion or endocytosis. Membrane fusion at the plasma membrane occurs after SARS-CoV-2 surface (S) protein activation by proteinase cleavage, including TMPRRS2, whereas fusion in the endocytic compartment requires the intracellular protease cathepsin L. The pathway and molecular players that govern endocytosis-mediated entry are, however, poorly characterized. Endocytosis of viruses can occur via different pathways, including clathrin-mediated endocytosis (CME), caveolar or lipid raft-mediated endocytosis, macropinocytosis and variations of these themes [3]. Some viruses are able to use more than one pathway, which introduces a further level of ambiguity into the classification of virus entry pathways.

Research on SARS-CoV-2 endocytic-mediated entry has been scant. A single report using a lentivirus system in ACE-2-overexpressing 293T cells suggests that SARS-CoV-2 endocytosis is dependent on clathrin and dynamin [4]. However, studies on the SARS-CoV-2 endocytic entry pathway in the context of a productive infection were missing at the beginning of our investigation.

ADP-ribosylation factor 6 (ARF6) is a multi-functional cellular protein. It directly activates lipid-modifying enzymes, such as phosphatidylinositol 4-phosphate 5-kinase, and it stimulates actin polymerization. ARF6-positive cargoes can be transported directly on microtubule filaments, and it also assists autophagy, particularly the initiation and autophagosome formation [5]. Because of its roles in endocytosis and trafficking, ARF6 is co-opted by several pathogens; bacteria (e.g. enteropathogenic and enterohaemorrhagic *Escherichia coli *, *Salmonella* Typhimurium, *Shigella *) and viruses (e.g. HIV-1, coxsackievirus A and B, Epstein–Barr virus). ARF6 was recently identified as a cellular interaction partner of the uridine-specific endoribonuclease Nsp15 of SARS-CoV-2 [6] and in a recent reanalysis of a RNA sequencing dataset of infected A549-ACE2 cells, ARF6 was involved in three of the four identified modules mediating host cell responses during SARS-CoV-2 infection, i.e. viral entry, regulation and signalling, and immune responses [7]. We therefore sought to determine the role of ARF6 in SARS-CoV-2 endocytosis and, more broadly, infection.

We first found that in the human hepatocarcinoma cell line, Huh-7 SARS-CoV-2 is resistant to camostat, a specific TMPRSS2 inhibitor, and hence SARS-CoV-2 entry mostly relies on endocytosis for infection. By genetic depletion and pharmacological inhibition, we further demonstrated that SARS-CoV-2 entry into Huh-7 cells is dynamin-independent but dependent on cholesterol and ARF6. This host factor is also important for infection in the more physiologically relevant models of human lung Calu3 cells and kidney organoids, suggesting that ARF6 might be an effective therapeutic target.

## Methods

### Cells, virus and compounds

Human hepatocarcinoma cells, Huh-7, and human lung adenocarcinoma cells, Calu3, were maintained at 37 °C and 5 % CO_2_ in Dulbecco’s modified Eagle’s medium (DMEM) (Gibco), supplemented with 10 % heat-inactivated foetal bovine serum (FBS), Hepes, nonessential amino acids, l-glutamine and 1× Pen–Strep (Gibco).

Kidney organoids were generated using human embryonic stem cells (UM77-2), as previously described [8]. SARS-CoV-2 was obtained through BEI Resources [SARS-related coronavirus 2, isolates USA-WA1/2020, NR-52281 and hCoV-19/USA/GA-EHC-2811C/2021 (omicron variant lineage B.1.1.529), NR-56481]. Lack of genetic drift of our viral stock was confirmed by deep sequencing. Viral titres were determined by 50% tissue culture infectious dose (TCID_50_) assays in Vero E6 cells (Reed and Muench method). All experiments using SARS-CoV-2 were performed at the University of Michigan under biosafety level 3 (BSL3) protocols in compliance with containment procedures in laboratories approved for use by the University of Michigan Institutional Biosafety Committee and Environment, Health and Safety.

Z-FA-FMK and camostat were kindly donated by the University of Michigan Center for Drug Repurposing, ß-methyl cyclodextrin and dynasore were purchased from Sigma Aldrich (cat. nos C4555 and D7693-5MG, respectively), NAV-2729 was obtained from Cayman Chemicals (cat. no. 26202) and remdesivir was purchased from MedChem Express (cat. no. HY-104077). Dynasore and NAV-2729 were used at 10 µM each, ß-methyl cyclodextrin at 2.5 µM and remdesivir at 1 µM.

### SARS-CoV-2 infection and quantification by immunofluorescence

Cells were seeded at 3000 cells/well (384-well plate, Perkin-Elmer, 6057300) or 10 000 cells/well (96-well plates, Costar, 3916) and allowed to adhere overnight. Compounds were then added to the cells and incubated for 2 h at 37 °C. Plates were then transferred to BSL3 containment and infected with SARS-CoV-2 WA1 or omicron at a multiplicity of infection (m.o.i.) of 1 (or an m.o.i. of 10 when indicated). In the case of infection with the omicron variant, pretreatment of the virus with 10 µM of porcine trypsin (MilliporeSigma, cat. no. T0303-1G) for 15 min at 37 °C was included. After 1 h of absorption on cells (final concentration of trypsin of 2 µM), the virus inoculum was removed and fresh media with inhibitors were added for indicated times. Uninfected and vehicle-treated infected cells were included as negative and positive controls, respectively. Cells were fixed with 4 % paraformaldehyde for 30 min at room temperature. Plates were then stained as previously described [9] by using anti-nucleocapsid protein (anti-N) SARS-CoV-2 antibody (Antibodies Online; cat. no. ABIN6952432) followed by staining with secondary antibody Alexa-647 (goat anti-mouse; Thermo Fisher; A21235) and DAPI for nuclei staining. Stained cells were acquired on Thermo Fisher CX5 high-content microscopes with a 20×/0.45 NA LUCPlan FLN objective. Laser autofocus was performed and 9 or 18 fields per well were imaged, covering ∼80 % of the well area. Images were analysed with Cell Profiler software and the percentage of infected cells (N-positive) was calculated on the total cell count (i.e. DAPI-positive).

### SARS-CoV-2 infection and quantification by TCID_50_ assay and RT-qPCR

Two hundred thousand Huh-7 or Huh-7 knock-out (KO) cells/well were seeded in 12-well plates. For Calu-3 cells, 100 000 cells/well were seeded in 24-well plates. The next day or at 80 % cell confluence, compounds were added to the cells and incubated for 2 h. Cells were then infected with SARS-CoV-2 WA1 at the indicated m.o.i. of 1 or 10 for 1 h at 37 °C. Inoculum was removed, cells were washed three times with phosphate-buffered saline (PBS) without magnesium and calcium (PBS−/−) and fresh medium was added. Cells were harvested 1 day post-infection (p.i.) and lysates were obtained by one cycle of freeze–thaw. For the kidney organoids, 3D spheres were collected in 1.5 ml tubes and infected with SARS-CoV-2 at an estimated m.o.i. of 1. After infection, 3D spheroids were washed three times with PBS−/− and gentle centrifugation (80 ***g*** for 2 min). A minimum of four 3D spheroids were transferred in a 96-well plate and medium with selected compounds was added to the cells. SARS-CoV-2 infection was quantified by TCID_50_ on preseeded Vero E6 cells (Reed and Muench method) or by RT-qPCR (IDT technologies, Charité kit) after RNA extraction with TRI reagent and Direct-Zol RNA miniprep kit (Zymo Research). For RT-qPCR, we followed the manufacturer’s directions of the iTaq Universal Probes One-Step RT-qPCR kit on an Applied Biosystems 7500 fast real-time PCR system in a reaction volume of 20 µl using previously described primers and probes (IDT cat. no. 10006804) with the following conditions: one cycle at 25 °C for 2 min, 55 °C for 10 min and 95 °C for 3 min each, followed by 40 cycles of 95 °C for 15 s and 58 °C for 30 s. To quantify genome equivalents, an external standard curve was established using a 10-fold serial dilution of a gBlock (IDT) containing the primer sequences of the SARS-CoV-2 E gene.

### Generation of CRISPR-KO cell lines

Individual guide RNA (gRNA) sequences (Table S1, available in the online version of this article) were cloned into BsmBI-digested lentiCRISPRv2 (Addgene #52961, a gift from Feng Zhang [10]) and resulting lentiviral stocks were prepared as previously described [11]. Huh-7 and Calu-3 cells were transduced with lentiviral stocks at an m.o.i. of ~1, treated 1 day later with puromycin (3 µg ml^−1^) until the death of control uninfected cells was complete, and passaged as needed to maintain logarithmic phase growth for 2–3 weeks. Cells were then either harvested for mRNA and protein preparations or infected with SARS-CoV-2 as described above.

### Attachment/internalization assay

Huh-7 or ARF6-KO cells were plated in 48-well plates at 100 000 cells per well and allowed to adhere overnight. The following day, NAV-2729 was added at the indicated concentration and incubated for 2 h. Following compound incubation, cells were infected with SARS-CoV-2 at an m.o.i. of 10 for 1 h at 4 °C to allow for viral binding. Cells were then washed three times with ice-cold PBS−/− to remove unbound virus and harvested in TRI reagent. Another set of treated-infected cells was transferred to 37 °C to allow for viral internalization. One hour after incubation, cells were treated with trypsin to remove bound-not internalized virus and after centrifugation at 500 ***g*** for 5 min, cells were harvested in TRI reagent. RNA was extracted by using the Direct-Zol RNA miniprep kit (Zymogen; R2052) and viral RNA was quantified by RT-qPCR as described above. The proportion of internalized virus was calculated over the bound virus.

## Results

### SARS-CoV-2 infection in Huh-7 is resistant to camostat treatment but sensitive to Z-FA-FMK

**Fig. 1.**
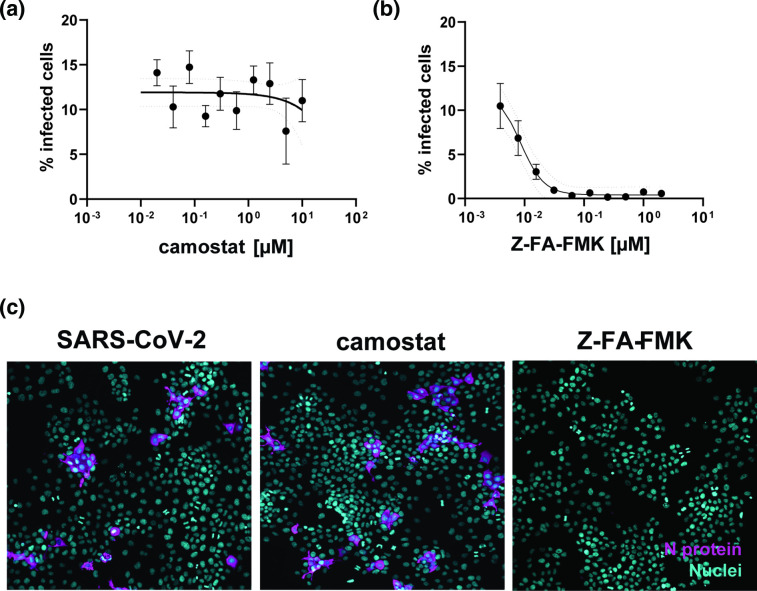
Huh-7 cells are resistant to the antiviral effect of camostat mesylate. (**a, b**) Dose–response curves of camostat mesylate (**a**) and the cathepsin B and L inhibitor Z-FA-FMK (**b**). Huh-7 cells were pretreated with compound and infected with SARS-CoV-2 at an m.o.i. of 1 for 1 h at 37 °C. At 48 h p.i., cells were fixed, permeabilized and stained with anti-N antibody. Cells were imaged with the high-content imaging platform CX5 and analysed with Cell Profiler for image segmentation. The % of infected cells was normalized to the non-treated infected condition. The graph represents the average sd of *n*=3 independent experiments with *n*=3 technical replicates, each. (**c**) Representative images of Huh-7 cells infected with SARS-CoV-2 or infected/treated with camostat (500 nM) and Z-FA-FMK (500 nM). N protein staining is represented in magenta and nuclei are in blue.

To investigate SARS-CoV-2 endocytosis, we screened a range of cell lines for the antiviral activity of camostat mesylate, a specific inhibitor of TMPRSS2 (data not shown). Infection readout was carried out with an imaging-based pipeline by using a SARS-CoV-2 anti-nucleocapsid (N) antibody to detect infected cells. Interestingly, in the human hepatocarcinoma cell line Huh-7, SARS-CoV-2 was completely resistant to the antiviral action of camostat (Fig. 1a). In contrast, treatment with the irreversible cysteine protease inhibitor Z-FA-FMK, an inhibitor of the intracellular protease cathepsin L, resulted in strong antiviral activity with EC_50_ of 11 nM (Fig. 1b) [9]. Representative images of SARS-CoV-2-infected Huh-7 in the presence of Z-FA-FMK and camostat are available in Fig. 1(c). These data suggest that SARS-CoV-2 entry into Huh-7 cells is likely mediated by endocytosis and not fusion at the plasma membrane. Therefore, Huh-7 cells represent a model cell line to mechanistically study SARS-CoV-2 endocytic entry.

### SARS-CoV-2 uptake into Huh-7 cells is dynamin-independent

**Fig. 2.**
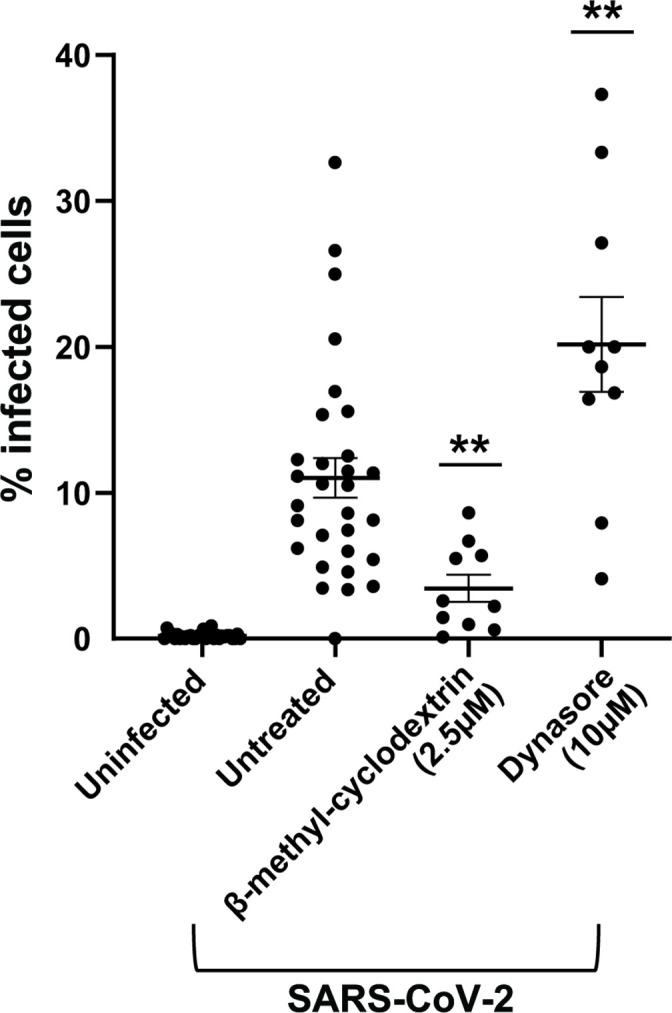
Screening of inhibitors of dynamin-dependent entry pathways in Huh-7 cells. Huh-7 cells were pretreated with compounds at the indicated concentration and infected with SARS-CoV-2 at an m.o.i. of 1 for 1 h at 37 °C. At 24 h p.i., cells were fixed, permeabilized, and stained with anti-N antibody. Cells were imaged with the high-content imaging platform CX5 and analysed with Cell Profiler for image segmentation. The graph represents the average sem of *n*=3 independent experiments with at least *n*=3 technical replicates each. Pairwise Student’s *t*-test was used to assess statistical significance; **, *P*-value <0.01.

A previous report with a lentivirus system in ACE-2-overexpressing 293T cells suggested that SARS-CoV-2 endocytosis is dependent on clathrin and dynamin [4]. We therefore determined whether dynamin governs SARS-CoV-2 entry into Huh-7 cells by using dynasore, a specific GTPase inhibitor of dynamin I, II and Drp1 (the mitochondrial dynamin) [12]. ß-methyl cyclodextrin, a cholesterol-removing agent that mostly blocks the formation of lipid rafts that are important for endocytosis, was used as a positive control since ACE2 is present in lipid rafts [13] and SARS-CoV entry is dependent on cholesterol [14]. Huh-7 cells were pretreated for 2 h prior to infection with selected compounds at non-toxic concentrations (Fig. S1) and infection with SARS-CoV-2 was quantified by N protein expression. As expected, treatment with ß-methyl cyclodextrin blocked viral infection. However, in contrast with previous findings, treatment with the specific dynamin inhibitor dynasore revealed a significant increase in the percentage of infected cells (Fig. 2). This observation points to a dynamin-independent but cholesterol-dependent endocytic uptake mechanism of SARS-CoV-2 into Huh-7 cells.

### Genetic perturbation of ARF6 results in a reduction in the percentage of infected cells

**Fig. 3.**
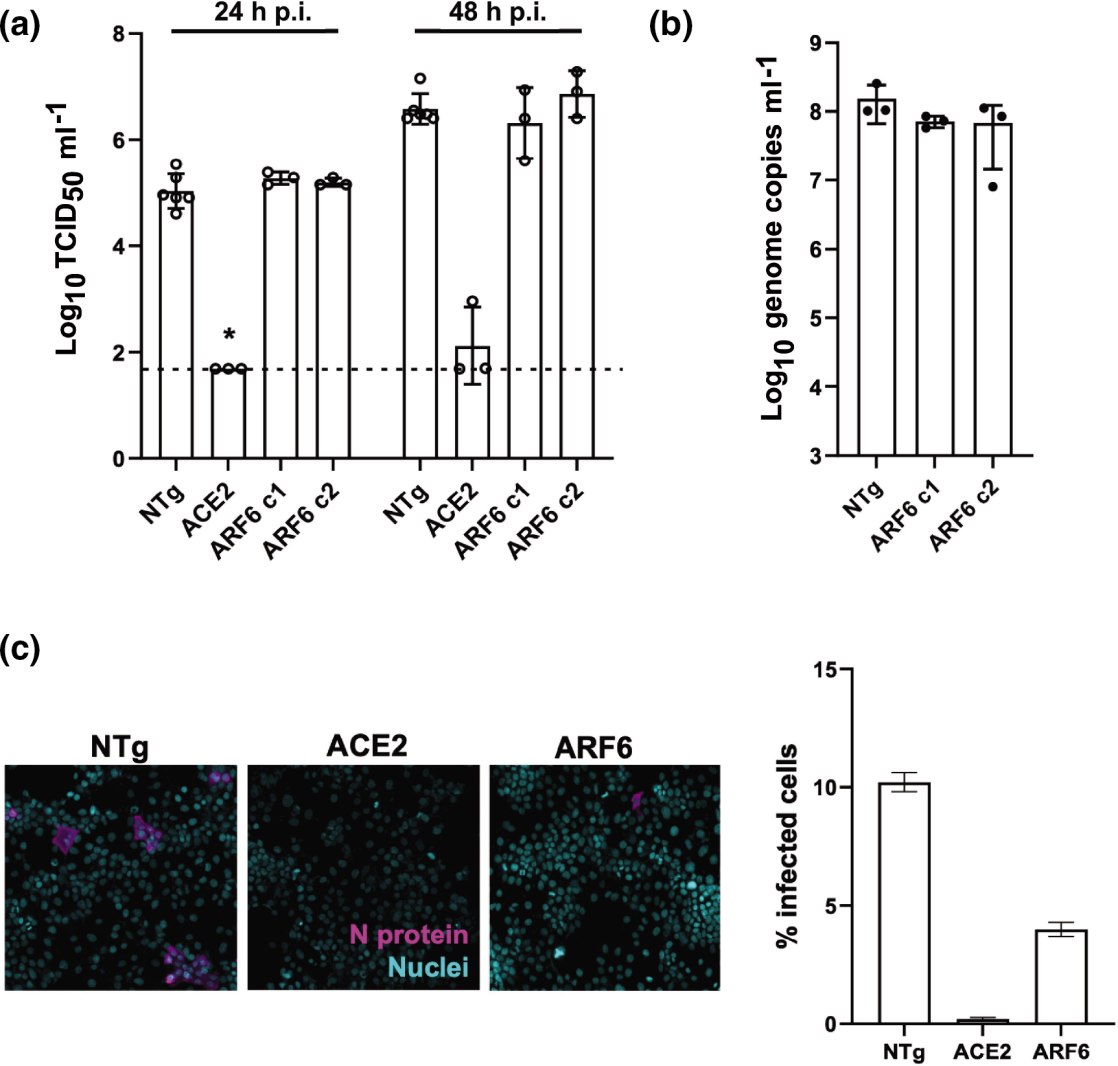
Genetic depletion of ARF6 does not affect viral titres but reduces the percentage of infected cells. (**a, b**) Viral titre determination of SARS-CoV-2-infected NTg, ACE2-KO and ARF6-KO Huh7 clones (m.o.i. of 1) by TCID_50_ ml^−1^ at 24 and 48 h p.i. (**a**) and by RT-qPCR at 24 h p.i. (**b**). ACE2-KO was used as a negative control of infection. Viral infectious titre was determined by TCID_50_ on Vero E6 cells. The dashed line represents the limit of detection of the TCID_50_ assay. (**c**) Cells were infected with SARS-CoV-2 at an m.o.i. of 10 for 24 h. Representative images of SARS-CoV-2-infected NTg and ARF6-KO-Huh7 clones are shown with N protein staining in magenta and nuclei in blue (DAPI). The graph represents the quantification of SARS-CoV-2-infected cells across independent experiments. Cells were imaged with the high-content imaging platform CX5 and analysed with Cell Profiler for image segmentation. The graph represents the average sem of at least *n*=3 independent experiments. Pairwise Student’s *t*-test was used to assess statistical significance; *, *P*-value <0.05

Dynamin-independent pathways are governed by Rho GTPase-activating protein 26 (GRAF-1), flotillin (FLOT-1) or ADP-ribosylation factor 6 (ARF6) [15]. Since ARF6 was recently described as mediating host cell responses during SARS-CoV-2 infection, we generated two polyclonal populations of CRISPR-targeted cells with gRNAs specific for ARF6 (clones 1 and 2). ARF6 depletion was confirmed by RT-qPCR, whereby the efficacy of knock out (KO) was measured as a percentage of mRNA target gene expression compared to the non-targeting guide (NTg) Huh-7 cell line (for clone 1, c1=39 % and clone 2, c2=56 %) and by Western blot (Fig. S2a). To test the efficacy of the CRISPR pipeline, an ACE2-KO Huh-7 line was generated in parallel as a control. Cells were infected with SARS-CoV-2 at an m.o.i. of 1 and harvested 1 and 2 days p.i. A combination of TCID_50_ assay and RT-qPCR were used to quantify viral infection and replication, respectively (Fig. 3a, b). No significant differences were detected by TCID_50_ between ARF6-KOand NTg cells, whereas infection was ablated in the control ACE2-KO Huh-7 cells (Fig. 3a). Consistent with that, viral replication in NTg and ARF6-KO cells was also similar (Fig. 3b). A kinetics of SARS-CoV-2 infection in NTg and ARF6-KO cells at an m.o.i. of 1 and 10 confirmed that there were no differences in viral titres by TCID_50_, and genome copies by RT-qPCR at 0 and 24 h p.i. (Fig. S3a, b). To independently validate these findings, an imaging-based readout for SARS-CoV-2 infection was used, since it enables analysis at the single cell level. Cells were infected with SARS-CoV-2 at the higher m.o.i. of 10 for 24 h (Fig. 3c). For ease of experimentation, only ARF6-KO c1, with lower expression of the target gene was used for this analysis. Representative images at 1 day p.i. and quantification are shown in Fig. 3(c). Intriguingly, we observed a significant decrease in the percentage of infected cells in ARF6-KO compared to the NTg cells, suggesting that ARF6 might be involved in SARS-CoV-2 entry (Fig. 3c). A subsequent dose–response study confirmed that N protein expression was significantly lower in SARS-CoV-2-infected ARF6-KO compared to the NTg cells at an m.o.i. of 10, but not at an m.o.i. of 1 (Fig. S3c). Collectively, these data suggest a role for ARF6 in SARS-CoV-2 infection in Huh-7.

### Pharmacological inhibition of ARF6 blocks SARS-CoV-2 internalization

**Fig. 4.**
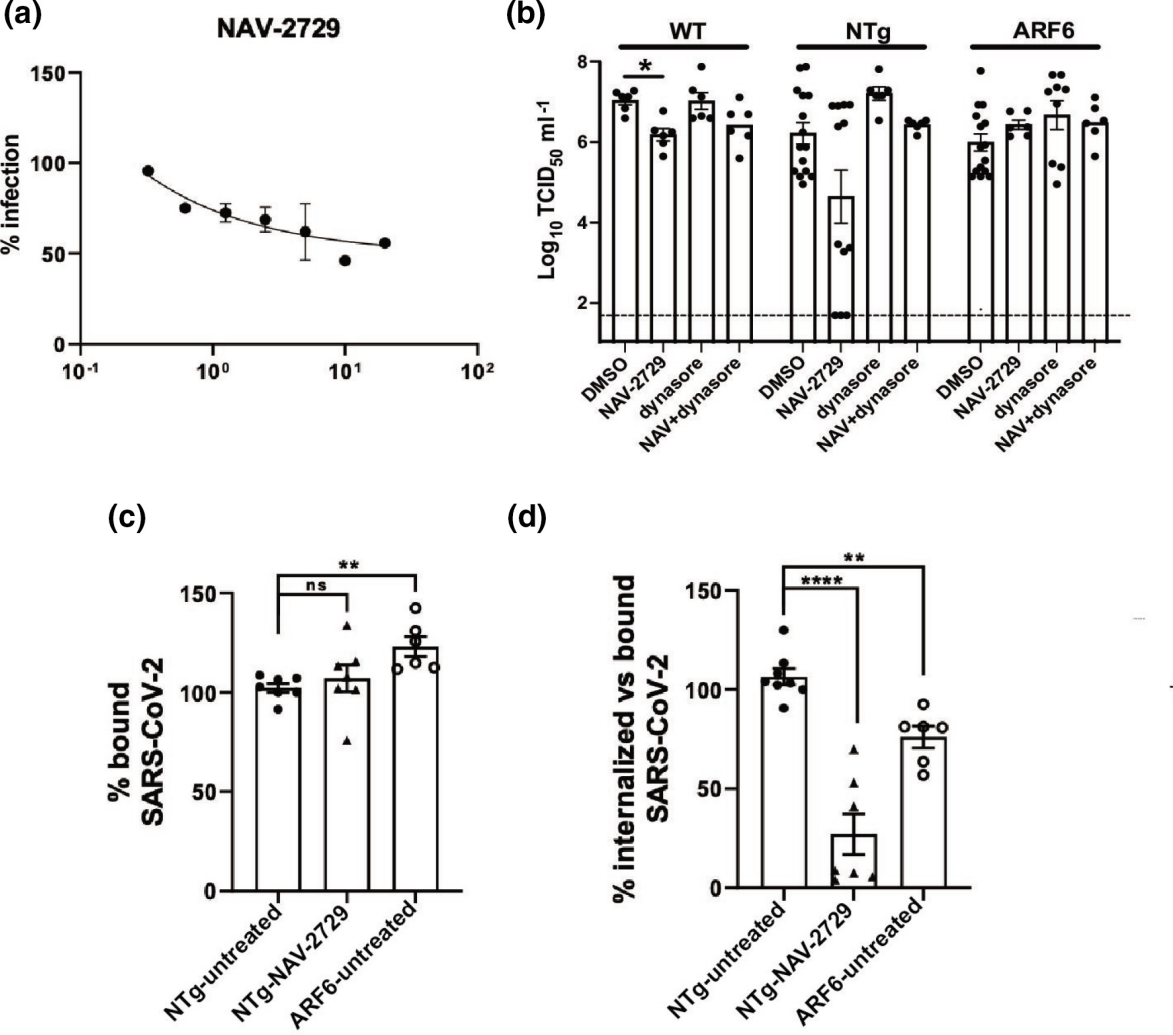
ARF6 is important during SARS-CoV-2 internalization in Huh-7 cells. (**a**) NAV-2729, an ARF6 inhibitor, was used to pretreat Huh-7 cells in a 384-well plate 2 h prior to infection with SARS-CoV-2 (m.o.i. of 1). Cells were harvested at 48 h p.i. and subjected to the imaging pipeline for viral quantification. Then 1 : 2 dose–response curves were developed in triplicate with three technical replicates each. (**b**) NTg, ARF6-KO and wild-type (WT) Huh-7 cells were pretreated with NAV-2927 (10 µM) and dynasore (10 µM) individually or in combination for 2 h before infection with SARS-CoV-2 (m.o.i. of 1). DMSO was used as vehicle control. Virus inoculum was removed 1 h p.i. and cells were incubated with fresh medium+/−compounds. Cells were harvested 24 h p.i. and viral titre was determined by TCID_50_ in Vero E6 cells. (**c, d**) NAV-2729 was used to pretreat Huh7 cells 2 h prior to infection with SARS-CoV-2 (m.o.i. of 10) on ice. NTg and ARF6-KO Huh7 cells were used as a control. One hour post-infection on ice, cells were washed twice and one set was harvested in TRI reagent for RNA extraction. The second set was incubated at 37 °C to allow viral internalization. One hour post-incubation, cells were treated with trypsin to remove bound, non-internalized virus and were harvested in TRI reagent for RNA extraction. Bound and internalized virus genome equivalents were determined by RT-qPCR: the graph in (c) shows the % of bound virus over the untreated control (NTg Huh-7), whereas the graph in (d) measures the internalized fraction of SARS-CoV-2 over the bound fraction. A statistical *t*-test was performed using GraphPad Prism with at least *n*=2 independent biological replicates and with two–three technical replicates each. ns, not significant; *,*P*-value <0.05; **, *P*-value <0.01; ****, *P*-value <0.001

ARF6 has been the object of extensive studies and specific small molecule inhibitors are available commercially. We therefore selected the inhibitor NAV-2729 to test its effect on SARS-CoV-2 infection. Huh-7 cells were pretreated with a 1 : 2 dilution series of compounds and infected with SARS-CoV-2 at an m.o.i. of 1. NAV-2729 showed a dose-dependent inhibition of infection (Fig. 4a), suggesting that ARF6 is an important factor for SARS-CoV-2 infection in Huh-7.

We next selected an efficacious non-toxic concentration of NAV-2729 (Fig. S1a) to confirm inhibition by TCID_50_ (Fig. 4b). Treatment with 10 µM NAV-2729 resulted in a~1 log reduction in viral infection in the NTg (although this did not reach statistical significance due to experimental variability) and in the wild-type (WT) Huh-7, included as a control to account for a differential effect of compounds in CRISPR-edited cells (Fig. 4b). No reduction in viral loads was observed in NAV-2729-treated ARF6-KO cells, as expected, emphasizing the specificity of the inhibitor under these conditions.

Given the redundancy of endocytic pathways and the previous report of dynamin-dependent SARS-CoV-2 pseudovirus entry [4], we next wanted to assess whether viral entry into ARF6-KO cells switched to a dynamin-dependent entry mechanism. Treatment of ARF6-KO cells with 10 µM dynasore did not result in changes to infection, further corroborating that dynamin is not involved in SARS-CoV-2 endocytosis in Huh-7 cells (Fig. 4b). However, double-treatment of WT, NTg or ARF6-KO cells with dynasore and NAV-2729 negated the inhibitory effect of NAV-2729 and resulted in similar viral titres to those for vehicle-treated cells.

To determine whether the NAV-2729 inhibitor worked at the level of viral internalization, an attachment/internalization assay was performed. Briefly, NTg cells were pretreated with NAV-2729 and infected with SARS-CoV-2 at an m.o.i. of 10. ARF6-KO cells were included as a control. One hour post-inoculation on ice, cells were washed and one set was harvested to quantify the virus attached at the cell surface by RT-qPCR. While treatment with NAV-2729 did not affect virus binding to NTg cells, ARF6-KO cells showed a significantly higher level of SARS-CoV-2 binding compared to NTg cells (Fig. 4c). The other batch of cells was incubated at 37 °C for 1 h to allow for viral internalization, and after unbound internalized virus was removed by treatment with trypsin, cells were harvested to quantify the internalized virus by RT-qPCR. To control for differences in SARS-CoV-2 binding between cells, we calculated the ratio of internalized versus bound virus (Fig. 4d). NAV-2729 reduced viral internalization by 75 % versus the more modest 25 % observed in the ARF6-KO, suggesting that ARF6 is a factor that governs endocytosis in Huh-7.

### ARF6 is an important host factor for SARS-CoV-2 infection in more physiologically relevant infection models

**Fig. 5.**
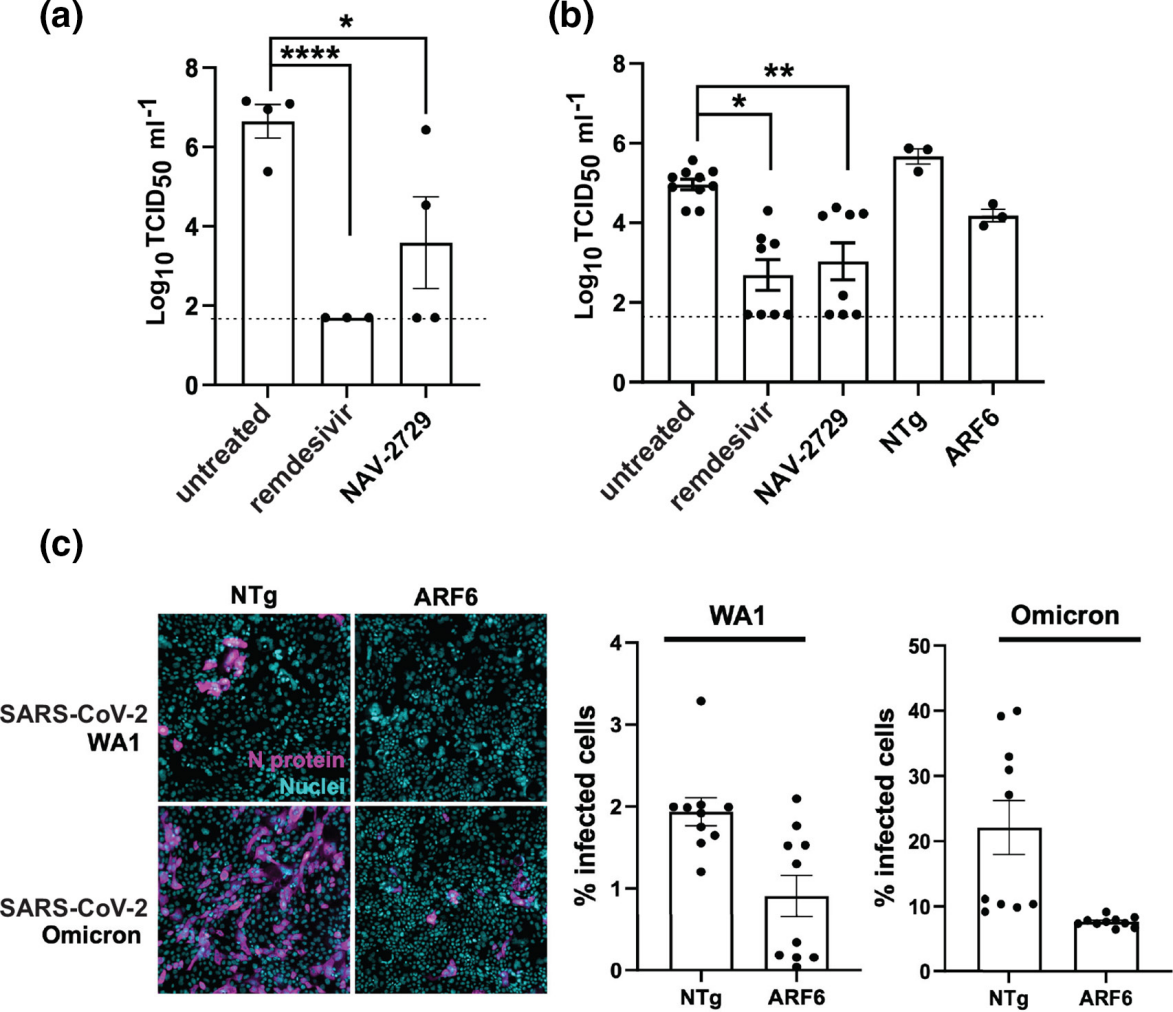
ARF6 is critical for SARS-CoV-2 infection in more physiologically relevant cell types. (**a**) Human kidney organoids were collected and resuspended in maintenance media. Groups of four organoids were infected with SARS-CoV-2 at an m.o.i. of 1. Organoids were washed twice and put in culture on a matrigel-coated plate for 48 h. Cells were then harvested and virus was quantified by TCID_50._ Each dot represents a technical replicate of *n*=2 biologically independent experiments. (**b**) Calu-3 cells and Calu-3 transduced with a non-targeting guide (NTg) or ARF6-targeting guide (ARF6) were seeded in 48-well plate and allowed to reach 80 % confluence. SARS-CoV-2 WA1 was used to infect cells at an m.o.i. of 1 for 24 h. Cells were then harvested and virus was quantified by TCID_50_. Each dot represents a technical replicate. (**c**) Representative images (left) and quantification (right) of Calu3 transduced with a non-targeting guide (NTg) or ARF6-targeting guide (ARF6) and infected with trypsin-activated SARS-CoV-2 WA1 or SARS-CoV-2 omicron (m.o.i. of 1) for 24 h. Cells were fixed, permeabilized and stained with anti-N primary antibody and anti-mouse AF647 secondary antibody (N protein, magenta) and Hoechst (nuclei, blue). Cells were imaged with the high-content imaging platform CX5 and analysed with Cell Profiler for image segmentation. Each dot represents the average of the % of infected cells over 20 fields of image per well. Each dot represents a technical replicate. Pairwise Student’s *t*-test was used to assess statistical significance. ns not significant; *, *P*-value <0.05; **,P-value<0.01; ****, *P*-value <0.001.

To test the importance of ARF6 in other cellular contexts, we used the ARF6 inhibitor NAV-2729 in more physiologically relevant models that are sensitive to camostat: human renal organoids and human lung Calu-3 cells [16]. In both models, treatment with a non-toxic concentration of NAV-2729 (Fig. S1b) resulted in a significant reduction in viral replication (Fig. 5a, b). The nucleoside analog remdesivir was used as a control.

In addition, we performed CRISPR/Cas9 knockout of ARF6 in Calu-3 cells (Fig. S2b) to corroborate the pharmacological studies with NAV-2729. Infection with SARS-CoV-2 at an m.o.i. of 1 was less efficient in the Calu3 line transduced with the ARF6-targeting guide (ARF6) than in Calu-3 cells transduced with a non-targeting RNA guide (NTg) (Fig. 5b), although the data did not reach statistical significance.

Lastly, to shed more light on the mechanism by which ARF6 assists SARS-CoV-2 replication in Calu-3 cells, we infected NTg and ARF6-KO Calu-3 cells with the SARS-CoV-2 omicron variant (lineage B.1.1.529). This variant has been demonstrated to rely primarily on endocytosis and not on membrane fusion for entry *in vitro* and *in vivo* [17]. Both virus isolates were pretreated with trypsin prior to infection to proteolytically cleave the S protein and bypass the need for cellular proteases [18]. Under these conditions, infection of NTg Calu-3 cells with SARS-CoV-2 omicron resulted in ~15 fold higher infection (as measured by % of N-positive cells at 24 h post-infection) than with the WA1 strain (Fig. 5c). Infection of ARF6-KO with both strains showed a significant reduction compared to NTg cells, suggesting that ARF6 might play a role in post-entry steps in Calu-3 cells. Overall, these results point to the importance of ARF6 in SARS-CoV-2 infection and to it being a putative therapeutic target against coronavirus infection.

## Discussion

In this study, we uncovered that ARF6 is a host factor during SARS-CoV-2 infection in cells of liver, lung and kidney origin. In addition, in Huh7 cells ARF6 inhibited SARS-CoV-2 uptake with a mechanism that is further dependent on cholesterol but independent of dynamin and by extension likely clathrin-independent. This is consistent with a prior study that excludes the involvement of dynamin, clathrin, caveolin and endophilin A2 for entry of SARS-CoV-2 [14] and with the entry pathway identified for SARS-CoV, which is cholesterol-dependent but clathrin- and caveolin-independent [19].

However, our finding differs from a study demonstrating the importance of clathrin in SARS-CoV-2 pseudovirus entry [4]. That study was performed with a lentivirus system in 293T cells and not in the context of authentic SARS-CoV-2 infection. Thus, differences in virus and/or cell type may alter the endocytic uptake pathway.

During the revision of this manuscript, Zhou *et al*. also published that ARF6 is an important host factor for SARS-CoV-2 entry [20]. Specifically, their study used an siRNA approach and SARS-CoV-2 pseudovirus to demonstrate a twofold reduction of viral entry in Huh-7 cells. This is consistent with the levels of reduction that we observe in the context of SARS-CoV-2 infection in ARF6-KO cells (by immunofluorescence) and upon pharmacological inhibition of ARF6 by the specific inhibitor NAV-2729. Our dose–response study (Fig. S3c) highlighted that the observed effect of ARF6 on infectivity in Huh-7 cells by immunofluorescence is only visible at a higher m.o.i., but that measurements of infectious virus titres become saturated at equivalent m.o.i., masking these differences.

Intriguingly, we observed that CRISPR-depleted cells exhibited a less pronounced phenotype as compared with those treated with the ARF6-specific inhibitor, NAV-2729. This could be due to several reasons. Because of the redundancy of endocytic pathways and lack of an important intracellular trafficking factor, prolonged passaging of the ARF6-KO cells and the potential incomplete activity of the guide RNA as indicated by the qPCR data might have led to compensatory mechanisms and a less uniform phenotype. In contrast, short-term treatment with a small molecule inhibitor at non-toxic concentrations does not affect or modify the physiology of the cell and reduces the impact of compensatory mechanisms and could thus be more relevant for the study of these redundant endocytic pathways. In addition, we cannot rule out that other targets of NAV-2729 may have additional impacts on SARS-CoV-2 infection [21].

In our investigation, we extended studies regarding the importance of ARF6 to the infection of other cell types – kidney organoids and Calu3 cells, a model of respiratory epithelial cells. In both systems, the ARF6 inhibitor, NAV-2729, showed a strong antiviral effect. These cell models support SARS-CoV-2 entry via membrane fusion [22]. Considering the multiple functions of ARF6 in intracellular membrane trafficking [5], we cannot rule out a role for ARF6 in the post-entry steps of the viral life cycle in these cell types, but additional studies are needed in the future to examine this possibility. Taken together, the data from liver, lung and kidney cells highlight a role for ARF6 during SARS-CoV-2 infection.

It is not known what proportion of SARS-CoV-2 virions enter via membrane fusion at the plasma membrane or endocytosis *in vivo*. SARS-CoV-2 prefers activation by TMPRSS2, but if the target cells express insufficient TMPRSS2, or if a virus–ACE2 complex does not encounter TMPRSS2, ACE2-bound virus is internalized via endocytosis [2]. TMPRSS2 is found in the gastrointestinal, respiratory and urogenital epithelium, but not in other extra-mucosal compartments (i.e. the heart, liver, kidneys, brain, spleen, and lymph nodes), where virus RNA or antigen is found in severe and deadly cases of COVID-19, suggesting that entry and replication of SARS-CoV-2 in these organs might be exclusively driven by endocytosis. It is also possible that SARS-CoV-2 entry occurs by plasma membrane fusion and endocytosis, regardless of the compartment. Therefore, it would be of interest to use NAV-2729 in a small animal model to investigate the *in vivo* relevance of the endocytic pathway and its importance in driving severe disease. Altogether, these data highlight ARF6 as a potential target in the development of therapeutics against COVID-19. Future studies focusing on investigational drugs targeting ARF6, such as Linsitinib, which has antiviral activity in a pseudovirus assay [7], could hold promise for the development of additional SARS-CoV-2 therapeutics.

## Supplementary Data

Supplementary material 1
