# Supplementary material for: ARF6 is a host factor for SARS-CoV-2 infection in vitro
Source: J Gen Virol. 2023 Jun 21;104(6):001868. doi: 10.1099/jgv.0.001868 (PMC10397720; doi:10.1099/jgv.0.001868)
Supplement: Supplementary material 1 [file jgv-104-1868-s001.pdf]

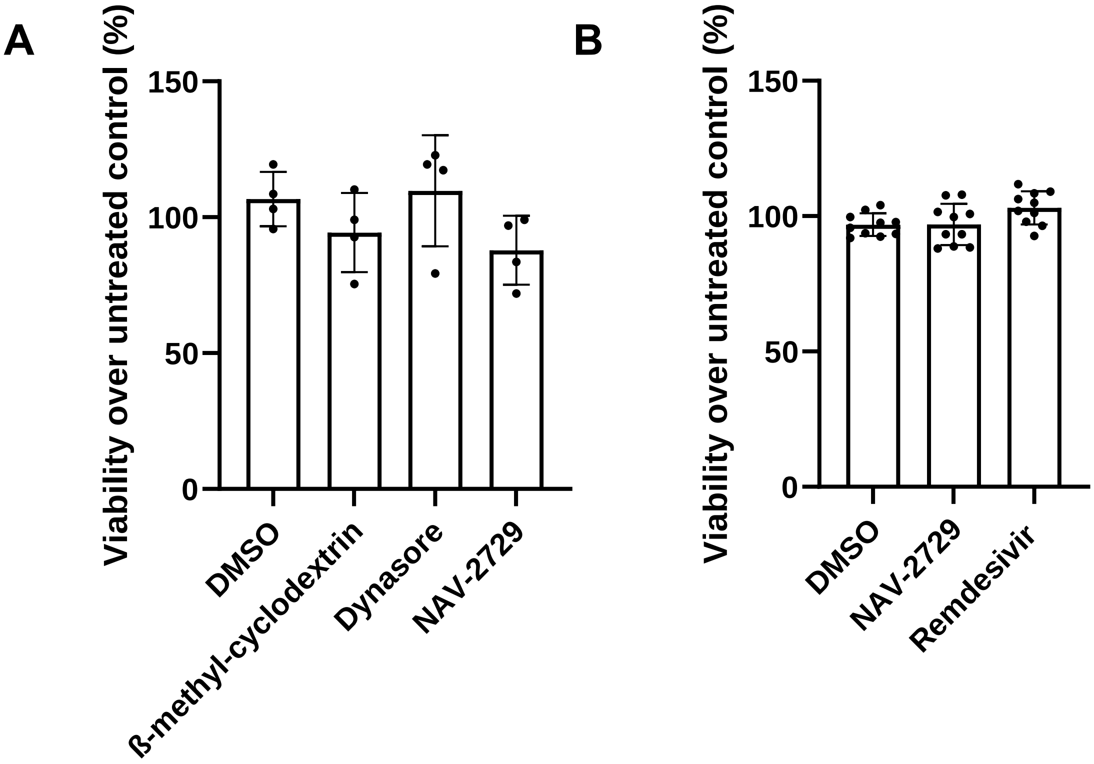

Supplementary figure 1: A-B) Toxicity assay of selected drugs by Resazurin assay in Huh-7 (panel A) and Calu-3 cells (panel B). Dynasore and NAV-2729 were tested at 10 μM each, β-methyl-cyclodextrin at 2.5 μM, and Remdesivir at 1 μM. DMSO was used as vehicle control.

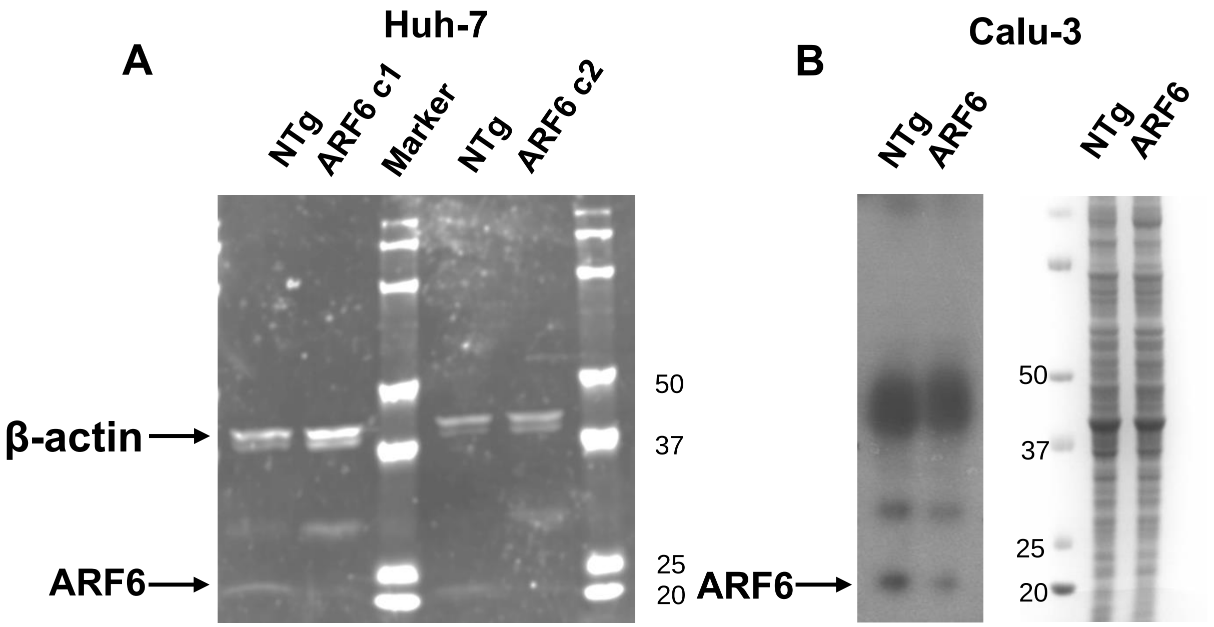

Supplementary figure 2: Western blot for ARF6 expression. A) Huh-7 lysates from non-targeting (NTg) and 2 clones of ARF6-knock-out (KO) preparations were obtained from cells seeded overnight in a 6-well plate and analyzed for ARF6 expression by Western blot. ß-actin was used as a loading control. Molecular weight marker (in kDa) is shown on the right. B) NTg and ARF6-KO Calu-3 cells were similarly obtained and analyzed by Western blot. As a loading control, a Coomassie blue staining of the gel was performed. Molecular weight marker (in kDa) is shown in the middle.

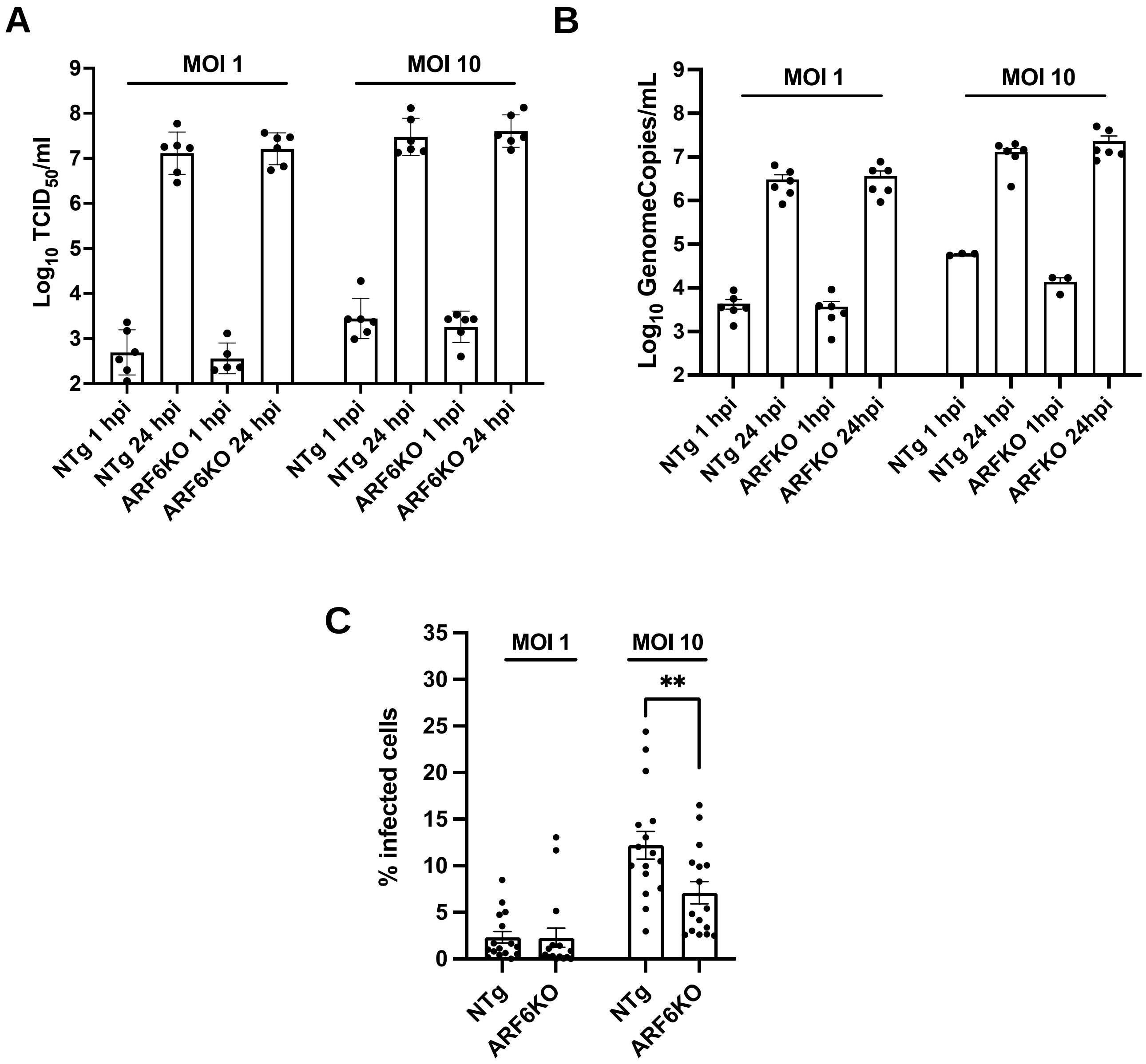

Supplementary figure 3: A-B) SARS-CoV-2 MOI dependency of infection in NTg and ARF6-KO Huh-7 cells. Readout was performed at 1 and 24 hpi by TCID$_{50}$ (**A**) and RT-qPCR (**B**), and at 24 hpi by immunofluorescence (**C**). Data are from two independent experiments with at least 3 technical replicates per experiment.

Supplementary Table 1: Sequences of sgRNAs for targeted CRISPR/Cas9 knockout.

Target gene(s)	gRNA	sgRNA
ARF6	1	CCTGGCGAGCCTCATCGATG
ARF6	2	CATTACTACACTGGGACCCA
ACE2	1	TACCAAGCAAATGAGCAGGG
Nontargeting	1	GTTCATTTCCAAGTCCGCTG

Supplementary Methods

Western blot. Cells were lysed in RIPA buffer (Pierce, 89900) with PhosSTOP (Roche, 04906845001) and cOmplete EDTA-free protease inhibitor cocktail (Roche, 11873580001) on ice. After vortexing to ensure a complete lysis, cells were centrifuged at 4°C, 14,000 × g for 15 min. The supernatant was mixed with β-mercaptoethanol-containing (10%) Laemmli buffer (Bio-Rad, cat nr. 1610747) at 3:1 ratio and denatured at 90°C for 10 min with a heat block. Samples were spun down before being loaded in a 4 to 20% Mini-Protean TGX gels (Bio-Rad, number 456-1096). Gels were run at 80 to 100 V for 45 min to 1 h and transferred to Immobilon-FL transfer membranes (IPFL00010) using semidry transfer at 10 V for 45 min. Membranes were blocked in 5% bovine serum albumin fat-free milk prepared in PBS containing 0.05% Tween 20 for a minimum of 2 hrs at room temperature. Membranes were incubated with primary antibodies (anti-ARF6 antibody (3A-1), Santa Cruz, cat nr. sc-7971 and anti-β–actin, 8H10D10, Cell Signaling, cat nr. 3700S) at 4°C by rocking overnight. Antibodies were washed off the membranes with four 15-min rocking periods using fresh changes of PBS. Secondary antibody incubation was done using fluorescent LI-COR antibodies (anti-rabbit IgG, cat. nr. 926-68071; and anti-mouse IgG, cat nr. 926-32210) or regular secondary antibody (anti-mouse IgG HRP, Thermo Fisher, A9044) diluted 1:10,000 in PBS for 1 hr at room temperature with gentle rocking. Membranes were washed with PBS as before and visualized using a LI-COR Odyssey Imager (Huh7 cells) or a regular imager after development with the Pierce™ ECL Plus Western Blotting Substrate (Thermo Fisher, cat nr. 32132X3) (Calu-3 cells).

Viability assay. Huh-7 cells or Calu-3 were seeded in a 96-well plate at 10,000 cells/well and allowed to adhere overnight. The next day, medium was replaced by medium with compounds or vehicle (DMSO, at the highest concentration used) and resazurin (Biotium, 30025-1) was added according to the manufacturer´s instruction. One day post treatment (to mimic infection condition), absorbance (590 nm) was measured with a Synergy™ HTX plate reader.
